# Prognostic and predictive role of *EGFR* pathway alterations in biliary cancer patients treated with chemotherapy and anti-EGFR

**DOI:** 10.1371/journal.pone.0191593

**Published:** 2018-01-19

**Authors:** Caterina Peraldo-Neia, Giuliana Cavalloni, Elisabetta Fenocchio, Celeste Cagnazzo, Loretta Gammaitoni, Stefano Cereda, Guglielmo Nasti, Maria Antonietta Satolli, Giuseppe Aprile, Michele Reni, Antonio Avallone, Rosella Spadi, Tiziana Venesio, Vittoria Martin, Claudio Doglioni, Milo Frattini, Massimo Aglietta, Francesco Leone

**Affiliations:** 1 Medical Oncology, Fondazione del Piemonte per l’Oncologia (FPO), IRCCS-Institute of Candiolo, Candiolo, Italy; 2 Department of Oncology, University of Turin, IRCCS-Institute of Candiolo, Candiolo, Italy; 3 Clinical Research Office, Fondazione del Piemonte per l’Oncologia (FPO), IRCCS-Institute of Candiolo, Candiolo, Italy; 4 Department of Medical Oncology IRCCS Ospedale San Raffaele, Milan, Italy; 5 Department of Abdominal Oncology, National Cancer Institute "G. Pascale", Naples, Italy; 6 Medical Oncology Division 1, University Hospital “Città della Salute e della Scienza”, Turin, Italy; 7 Department of Oncology, San Bortolo Hospital ULSS8—East District, Vicenza, Italy; 8 Pathology Unit, Fondazione del Piemonte per l’Oncologia (FPO), IRCCS-Institute of Candiolo, Candiolo, Italy; 9 Institute of Pathology, Locarno, Switzerland; 10 Pathology Unit, IRCCS Ospedale San Raffaele, Milan, Italy; University of Crete, GREECE

## Abstract

The association of anti-EGFR to gemcitabine and oxaliplatin (GEMOX) chemotherapy did not improve survival in biliary tract carcinoma (BTC) patients. Multiple mechanisms might be involved in the resistance to anti-EGFR. Here, we explored the mutation profile of *EGFR* extracellular domain (ECD), of tyrosine kinase domain (TKD), and its amplification status. *EGFR* mutational status of exons 12, 18–21 was analyzed in 57 tumors by Sanger sequencing. EGFR amplification was evaluated in 37 tumors by Fluorescent In Situ Hybridization (FISH). Kaplan-Meier curves were calculated using the log-rank test. Six patients had mutations in exon 12 of *EGFR* ECD and 7 in *EGFR* TKD. Neither *EGFR* ECD nor TKD mutations affected progression free survival (PFS) or overall survival (OS) in the entire population. In the panitumumab plus GEMOX (P-GEMOX) arm, ECD mutated patients had a worse OS, while *EGFR* TKD mutated patients had a trend towards shorter PFS and OS. Overall, the presence of mutations in *EGFR* or in its transducers did not affect PFS or OS, while the extrahepatic cholangiocarcinoma (ECC) mutated patients had a worse prognosis compared to WT. Nineteen out of 37 tumors were *EGFR* amplified, but the amplification did not correlate with survival. ECC *EGFR* amplified patients had improved OS, whereas the amplification significantly correlated with poor PFS (p = 0.03) in gallbladder carcinoma patients. The high molecular heterogeneity is a predominant feature of BTC: the alterations found in this work seem to have a prognostic impact rather than a predictive role towards anti-EGFR therapy.

## Introduction

Different strategies aimed at inhibiting EGFR with small molecules (erlotinib and gefitinib) or with monoclonal antibodies (cetuximab and panitumumab) have been developed over the years in many cancer types [1–6]. Panitumumab (Vectibix, Amgen), a fully human antibody directed against EGFR, was initially approved in *KRAS* wild type (WT) metastatic colorectal cancer (mCRC) patients refractory to previous chemotherapy [7, 8]. In biliary tract carcinoma (BTC), preclinical evidence of antitumor activity [9] and the lack of compelling therapies suggested that the combination of standard chemotherapy and EGFR inhibitors could be an attractive option to improve patient outcome [10, 11]. The randomized, open-label, phase II Vecti-BIL trial compared the efficacy of gemcitabine and oxaliplatin (GEMOX) chemotherapy with or without panitumumab (P) in *KRAS* WT advanced BTC (Clinical Gov Identifier NCT01389414). The study, which enrolled and stratified intrahepatic cholangiocarcinoma (ICC) and extrahepatic cholangiocarcinoma (ECC) including gallbladder carcinoma (GBC), revealed that the addition of panitumumab to the standard chemotherapy did not improve progression free survival (PFS), which was 5.3 months in experimental arm and 4.4 months in control arm. No differences were observed in overall survival (OS), being of 9.9 with GEMOX and 10.2 months with P-GEMOX [12]. Hence, we concluded that *KRAS* WT status was not sufficient to select patients who can achieve tumor response to anti-EGFR therapies. Over the years, the phenomenon of resistance to anti-EGFR therapies has been deeply studied, in particular in CRC. The panel of potential drivers of resistance was expanded and *KRAS* exons 3–4, in addition to exon 2, *NRAS*, *PIK3CA*, and *BRAF* analyses were introduced in the clinical practice [13–15]. Thus, we retrospectively analyzed the mutational status of these genes in patients enrolled in the Vecti-BIL study and we found that the presence of these mutations did not affect the response to treatments. Recently, new mechanisms of resistance to anti-EGFR antibodies have been recognized in mutations of *EGFR* exon 12 of the extracellular domain (ECD); in CRC it was demonstrated that they prevented the correct binding of anti-EGFR, reducing their activity [16]. Moreover, even if controversial, *EGFR* amplification seemed to be a predictive marker of prognosis and response to the anti-EGFR therapies in CRC [15, 17]. *EGFR* amplification was also described in BTC [18, 19], but its prognostic role is unknown. Overall, in both arms of the Vecti-BIL trial, there was a broad range of PFS and OS: in the experimental arm, PFS ranged from 1.1 to 21.3 months and OS from 2.7 to 34.9 months, while in the control arm PFS ranged between 1.1 to 15.4 months, and OS between 1.1 and 31.7 months.

Here, we extended the molecular analyses to the *EGFR* ECD and TKD mutation profiling, and to the *EGFR* amplification status to explain these differences, and to correlate them to the arm of treatment.

## Materials and methods

### Patients

The Vecti-BIL trial (ClinicalTrials.gov Identifier: NCT01389414) enrolled 89 BTC patients selected for the absence of *KRAS* exon 2 mutations. Forty-five patients were assigned to receive GEMOX in association with panitumumab (ARM-A) and 44 patients GEMOX alone (ARM-B). All patients enrolled in the study have signed the Independent Ethical Committees (IEC) informed consent, which provided the authorization to perform molecular analyses of *EGFR* status and of its principle transducers on archival tumor tissues. The study was performed in accordance with the Declaration of Helsinki and was approved by the “Comitato Etico San Luigi Gonzaga di Orbassano”. Of 89 patients enrolled in the study, 57 had formalin fixed paraffin embedded tissues (FFPE blocks or tissues slides) available for molecular analyses.

### Mutational analysis

For all the specimens, the tumor areas, identified by a pathologist, were scraped from two tissue slides (5 micron thick). DNA was then extracted by using Qiamp DNA FFPE mini kit (Qiagen s.r.l. Milano, Italy) following manufacturers’ instruction. Briefly, For DNA extraction, tissues were deparaffinized by xylene, rehydrated by ethanol, lysed with appropriate buffer and proteinase K at 56°C for one hour and subsequently for another hour at 90°C. Samples were then transferred on columns, washed with different buffers and then eluted in nuclease free water. DNA quantity was evaluated by Nanodrop (ThermoScientific Italia, Monza, Italy). The integrity of DNA was tested by a specific PCR for the housekeeping gene *GAPDH*. The *EGFR* exon 12 was amplified using nested PCR [16]. Exons 18–21 of *EGFR* were amplified by nested PCR using primers and conditions already described [20]. PCR products were purified using The Wizard SV Gel and PCR Clean-Up System kit (Promega Italia, Milano Italy). Each exon was sequenced using the BigDye Terminator Cycle sequence following the PE Applied Biosystem strategy and Applied Biosystems ABI PRISM3100 DNA Sequencer (Applied Biosystem, Forster City, CA). Sense and antisense sequences were obtained by using forward and reverse internal primers, respectively. All mutations were confirmed by performing two independent PCR amplifications.

### Fluorescent *in situ* hybridization (FISH) analysis

*EGFR* gene status evaluation was performed by FISH on 3-μm thick tissue sections. Dual-color FISH assay was performed using LSI *EGFR/CEP7* probes (Vysis, Abbott Laboratories, USA) following the manufacturer’s instructions already described [21]. Fluorescent *in situ* hybridization signals were evaluated with a Zeiss Axioscope (Carl Zeiss SPA, Italy). Tumors with ≥4 copies of *EGFR* or gene amplification in ≥40% of cells were classified as FISH+.

### Statistical analysis

KaplanMeier survival curves were calculated using the Graph Pad Prism 6 software. Molecular findings (mutational status of exon 12, 18–21 of *EGFR*, its transducers *KRAS*, *NRAS*, *BRAF*, *PIK3CA*,and FISH) were correlated with PFS an OS in the entire study population and separately in the two arms of treatment. The Cox proportional hazards regression model was used to identify prognostic factors. PFS and OS were calculated using Kaplan-Meier estimation and examined by log-rank test. The association between the radiological response and molecular findings was analyzed using the two tailed Chi-Square test (C.I. 95%). A p-value less than 0.05 was considered as statistically significant.

## Results

### Mutational status of *EGFR* extracellular domain (ECD) and tyrosine kinase domain (TKD)

**Fig 1:**
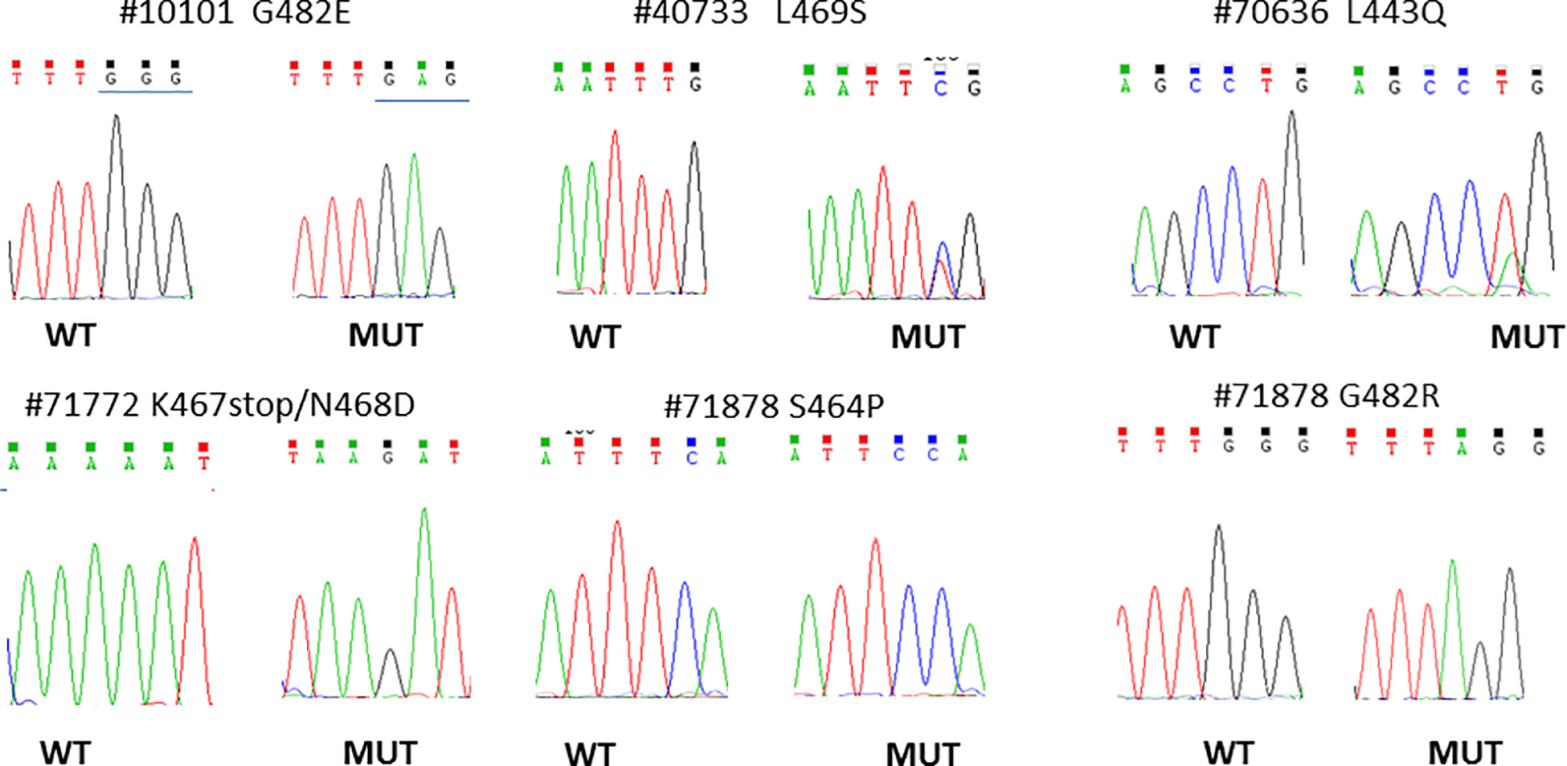
Representative electropherograms of *EGFR* ECD mutated samples.

**Fig 2:**
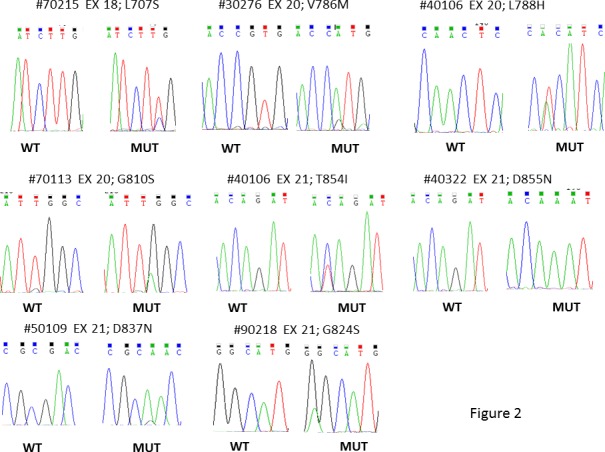
Representative electropherograms of *EGFR* TKD mutated samples.

Six out of 57 patients (10.5%) displayed mutations in *EGFR* ECD (exon 12). Four patients belonged to the ARM-A and two patients to the ARM-B. **Fig 1**shows the electropherograms of *EGFR* ECD (exon 12) mutated samples. In the *EGFR* TKD, we found mutations of exons 18–21 in 7 out of 57 samples (12.3%). In detail, exon 18 was mutated in one patient, exon 20 was mutated in 3 patients, and exon 21 was mutated in 4 patients; no mutations were found in exon 19. **Fig 2**showed the electropherograms of *EGFR* TKD. **S1 Table** summarizes the mutations found, some of which have already been described in literature. In our previous work we described 3 patients with *BRAF* V600E mutations, 2 with *NRAS* mutations (A146S and Q61R), and 2 with *PIK3CA* E545K mutations [12]. One patient, the 71772, which harbored a *PIK3CA* mutation, was also mutated in *EGFR* ECD. Overall, we found that 18 out of 57 patients (31.6%) had mutations in *EGFR* or in its transducers. The mutations were homogeneously distributed between the two arms of treatment (8 patients in P-GEMOX arm, and 10 patients in GEMOX arm). Nine out of 18 mutated tumors (50%) were ICC, while 3 were ECC (16.7%), and 6 were GBC (33.3%).

### *EGFR* ECD mutations are associated with a worsening survival in subgroups

**Fig 3:**
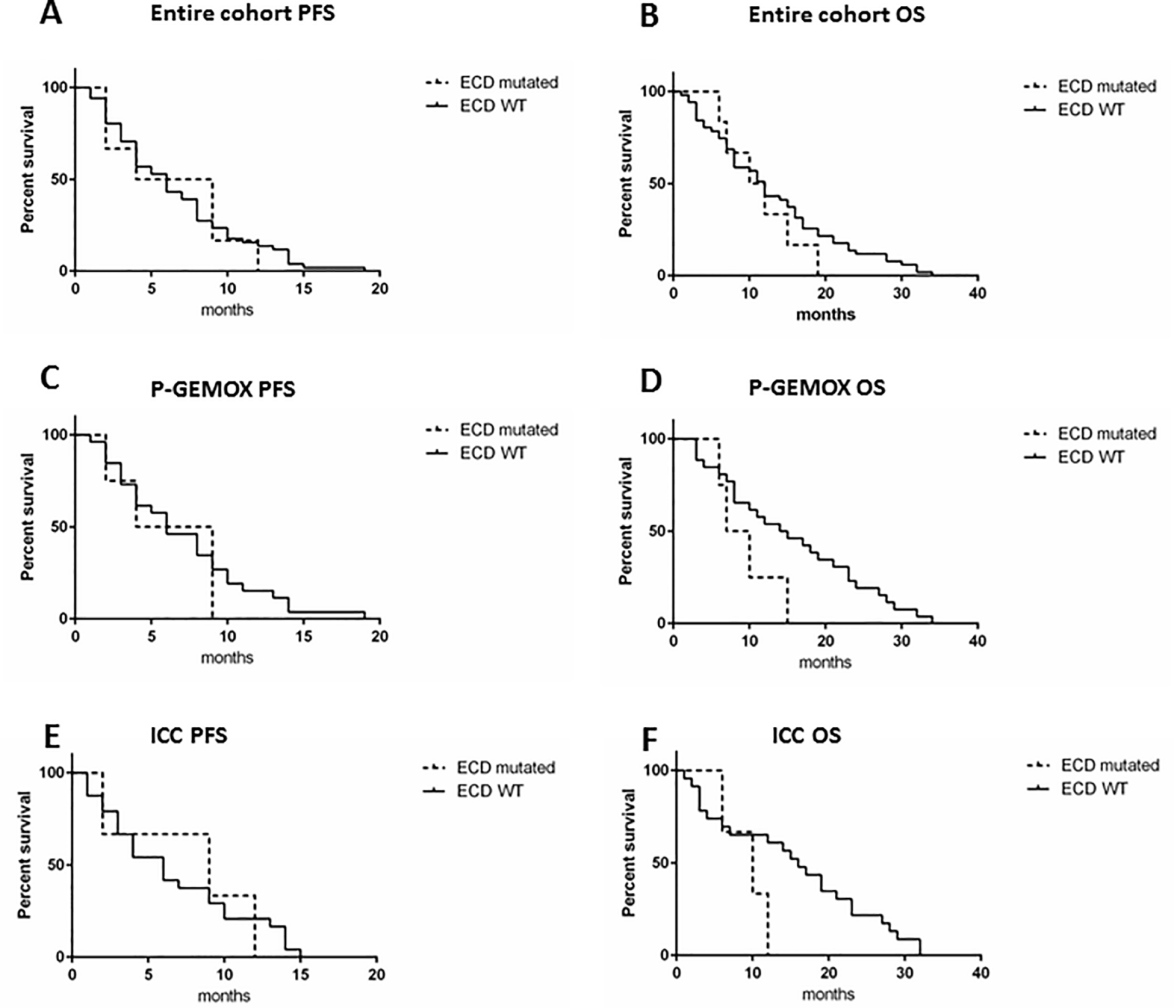
KaplanMeier survival curves in ECD mutated vs WT patients. A-B) association between the presence of ECD mutations and PFS and OS respectively, in the entire cohort of the Vecti-BIL study. C-D) association between the presence of ECD mutations and PFS and OS respectively, in P-GEMOX treated patients. E-F) association between the presence of ECD mutations and PFS and OS respectively, in ICC patients.

Analyzing all the 57 patients, independently from the treatment, we found that the presence of *EGFR* ECD mutations did not significantly affect PFS (p = 0.81) and OS (p = 0.44) (**Fig 3A and 3B**). In P-GEMOX arm, patients with *EGFR* ECD mutations had worse OS, even if not significantly (p = 0.1), while the PFS was not affected (p = 0.6) (**Fig 3C and 3D**). In the ICC subgroup, *EGFR* ECD mutated patients had impaired OS compared to WT patients (p = 0.14), but similar PFS (p = 0.92) (**Fig 3E and 3F**). Due to the small number of mutated patients, the analysis was not conducted for the GEMOX arm and for the other site subgroups.

### *EGFR* TKD mutations have a predictive negative role in P-GEMOX treated patients

**Fig 4:**
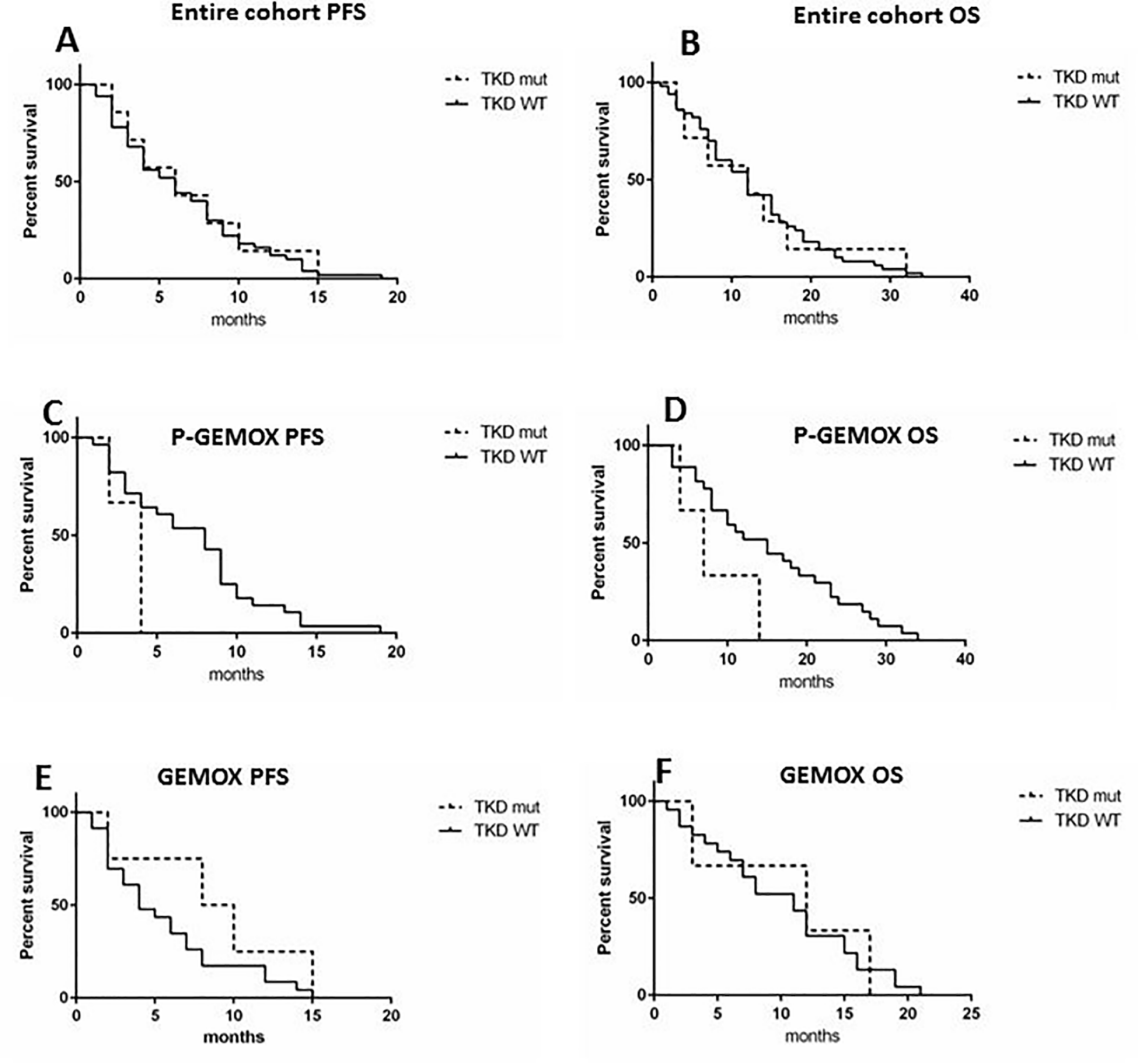
Kaplan-Meier survival curves in TKD mutated vs WT patients. A-B) Association between the presence of TKD mutations and PFS and OS, respectively, in the entire cohort of the Vecti-BIL study. C-D) Association between the presence of TKD mutations and PFS and OS, respectively, in P-GEMOX treated patients. E-F) association between the presence of TKD mutations and PFS and OS, respectively, in GEMOX treated patients.

The survival analysis of *EGFR* TKD mutated versus WT patients in the entire cohort of the study did not reveal any difference in terms of PFS (p = 0.75) and OS (p = 1.0) (**Fig 4A and 4B**). Even though, by analyzing P-GEMOX treated patients, we found that *EGFR* TKD mutations caused a trend towards decrease of PFS (p = 0.06) and OS (p = 0.06) (**Fig 4C and 4D**). On the contrary, *EGFR* TKD mutated patients treated with GEMOX had a better PFS (p = 0.2), while no differences in OS was observed (p = 0.9) (**Fig 4E and 4F**). For GBC patients, the presence of *EGFR* TKD mutations did not affect the PFS or OS (data not shown). Due to the small number of mutated patients, the analysis was not conducted for the other site subgroups.

### Wild-type status of *EGFR* and its transducers is not associated with efficacy of panitumumab

**Fig 5:**
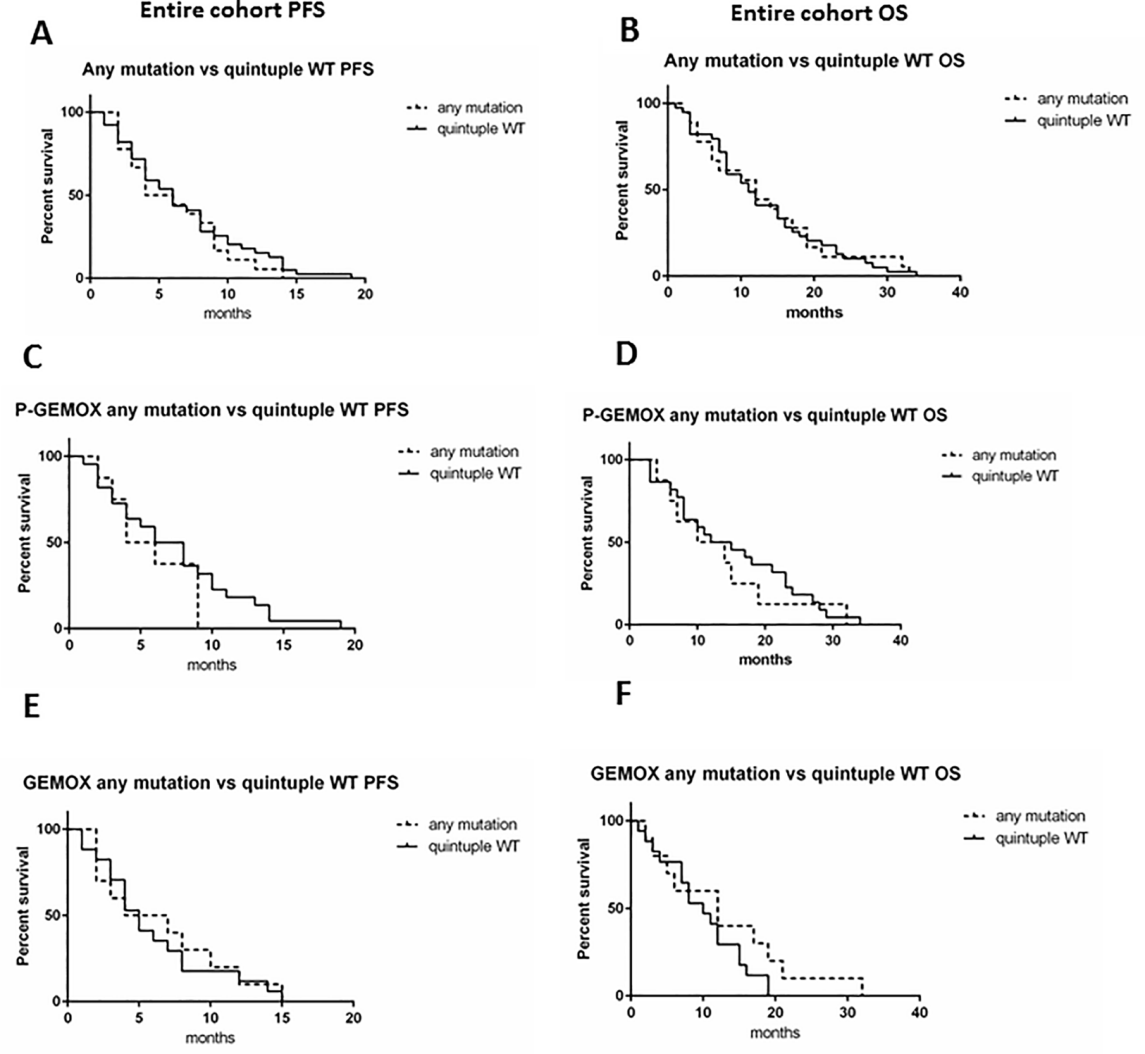
Kaplan-Meier survival curves in *EGFR* or its transducers mutated vs WT patients. A-B) association between the presence of any mutation and PFS and OS, respectively, in the entire cohort of the Vecti-BIL study. C-D) association between the presence of any mutation and PFS and OS, respectively, in P-GEMOX treated patients. E-F) association between the presence of any mutation and PFS and OS, respectively, in GEMOX treated patients. Quintuple WT (absence of *EGFR*, *KRAS*, *NRAS*, *BRAF*, or *PIK3CA* mutations).

We analyzed if the presence of any mutation affecting *EGFR* and its main transducers (*KRAS*, *NRAS*, *BRAF*, *PIK3CA*) could influence survival in BTC patients. As shown in **Fig 5A and 5B**, PFS and OS were not impaired by the presence of mutations in *EGFR* and/or its transducers in the entire cohort (p = 0.49 and p = 0.93, respectively) as well as considering the two arms of treatment (**Fig 5C–5F**) (P-GEMOX; PFS: p = 0.29; OS: p = 0.6. GEMOX; PFS: p = 0.76; OS: p = 0.19). Then, we stratified patients according to the site subgroup and we found that ECC patients harboring mutations had a lower survival rates, even if not significantly, compared to WT patients (PFS: p = 0.12; OS: p = 0.17) (**S1C and S1D Fig**). No differences in terms of PFS and OS were evidenced in ICC (p = 0.57 and p = 0.25, respectively, **S1A and S1B Fig**) and GBC patients (p = 0.79 and p = 0.87, respectively, **S1E and S1F Fig**).

### *EGFR* amplification does not affect patient survival

**Fig 6:**
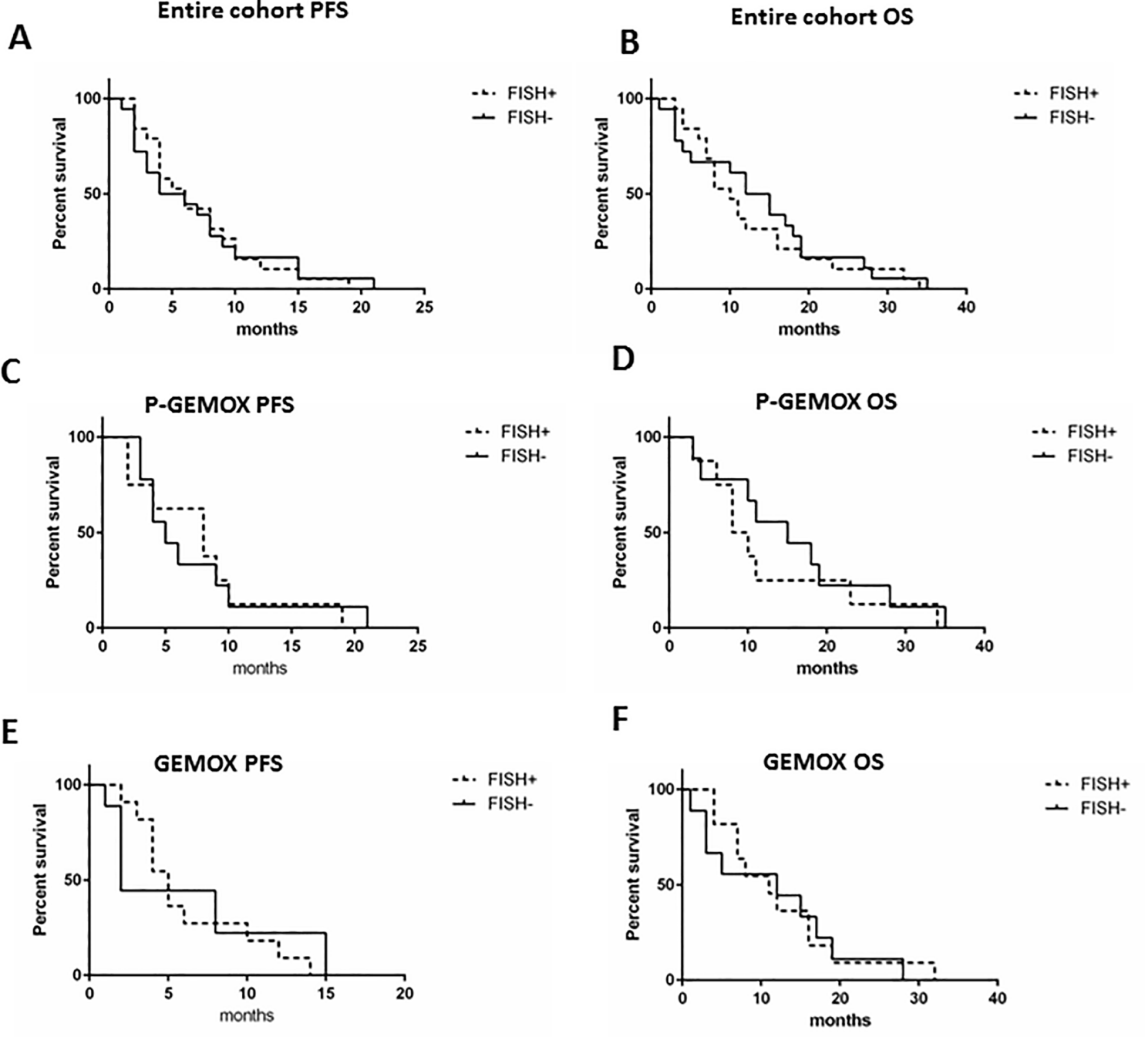
Kaplan-Meier survival curves in FISH+ or FISH- patients. A-B) Association between *EGFR* amplification (FISH+) and PFS and OS, respectively, in the entire cohort of the Vecti-BIL study. C-D) association between *EGFR* amplification (FISH+) and PFS and OS, respectively, in P-GEMOX treated patients. E-F) association between *EGFR* amplification (FISH+) and PFS and OS, respectively, in GEMOX patients.

**Fig 7:**
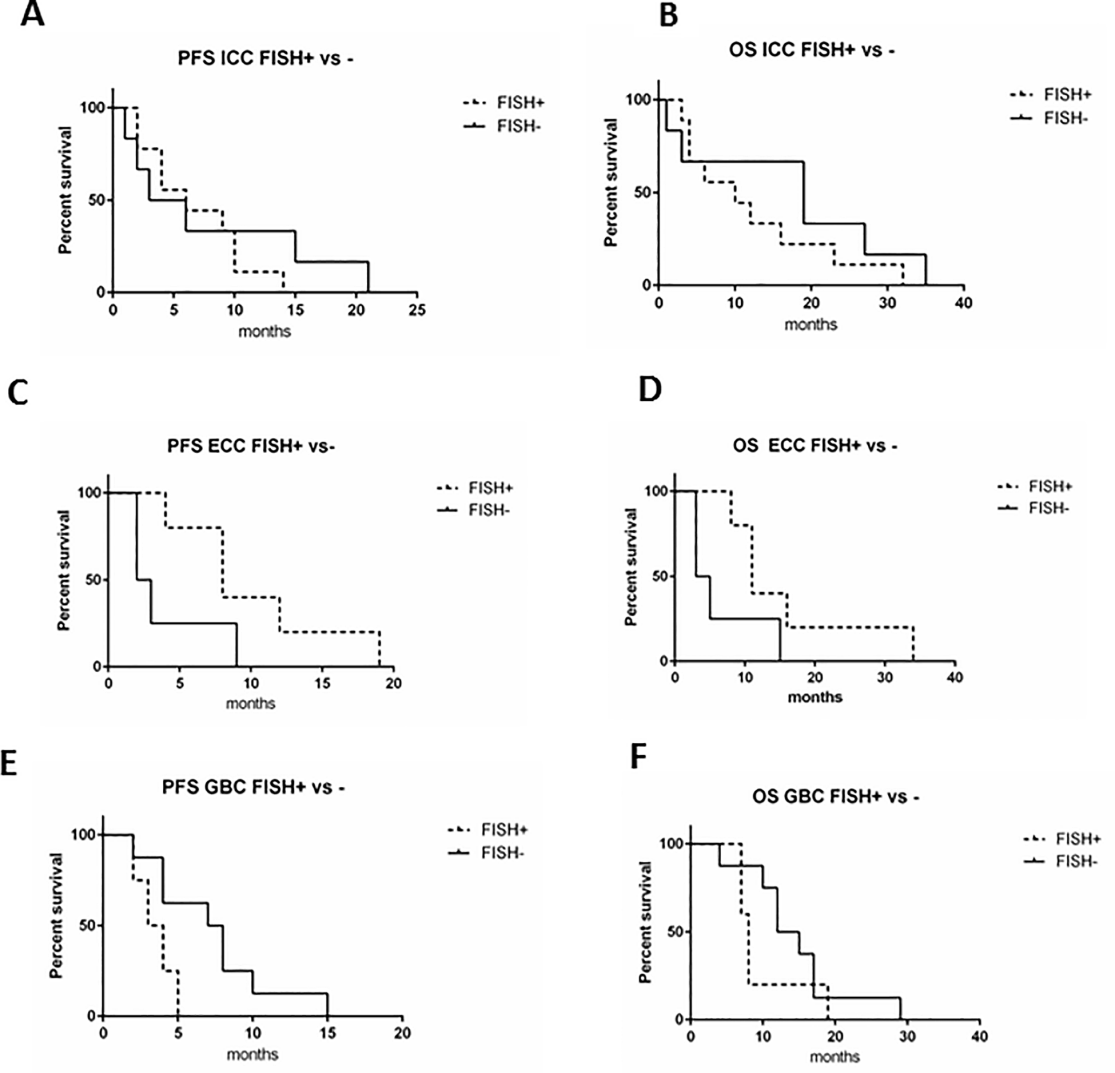
Kaplan-Meier survival curves in FISH+ versus FISH- patients. A-B) Association between *EGFR* amplification (FISH+) and PFS and OS, respectively, in ICC patients. C-D) Association *EGFR* amplification (FISH+) and PFS and OS, respectively, in ECC patients. E-F) Association between *EGFR* amplification (FISH+) and PFS and OS, respectively, in GBC patients.

FISH analysis was conducted in 37 tumor samples. Due to the inadequate tissue preservation, the other tumor samples resulted unsuitable for this test. The analysis revealed that 19 tumors (51.4%) had amplification of *EGFR* (FISH+), while the remaining 18 were classified as negative (FISH-). Among the FISH+ tumors, 8 belonged to ARM-A and 11 to ARM-B of treatment. Nine out of 19 FISH+ tumors were ICC, five were ECC and five GBC. Considering all the 37 patients analyzed, PFS and OS were comparable in *EGFR* FISH+ and *EGFR* FISH- patients (PFS: p = 0.98; OS: p = 0.59) (**Fig 6A and 6B**). Similar results were obtained analyzing the two arms of treatment separately. **Fig 6C and 6D** shows the PFS and OS survival curves of P-GEMOX treated patients (p = 0.97 and p = 0.45, respectively) and **Fig 6E and 6F** represents the PFS and OS survival curves of GEMOX-treated patients (p = 0.68 and p = 0.46, respectively). Further, we stratified patients according to the site subgroup; as shown in **Fig 7**, ECC *EGFR* FISH+ patients had a better PFS (p = 0.07) and OS (p = 0.08) compared to FISH- patients (**Fig 7C and 7D**), even if not significantly. On the contrary, in GBC patients, FISH+ was associated to a PFS significantly worse (p = 0.03) and an OS slightly reduced (p = 0.23, **Fig 7E and 7F**). Finally, in ICC, FISH+ did not affect PFS (p = 0.54) and OS (p = 0.33, **Fig 7A and 7B**).

## Discussion

The role of EGFR in cholangiocarcinogenesis is well documented. Its high expression as well as the low percentage of mutations in its downstream effectors suggested that it could be a suitable target for molecular therapy. Even though, these premises did not reflect the success obtained in mCRC and now the scientific community agreed about the marginal role of anti-EGFR therapies in BTC. The lack of efficacy obtained by the addition of panitumumab to standard chemotherapy demonstrated in the Vecti-BIL trial confirmed previous results available in literature [22–24]. The difference in median PFS and OS obtained in the two arms of treatment were not statistically different, but each arm displayed a broad range of PFS and OS, suggesting that both prognostic and predictive factors may have affected the results. The predefined collection of tumor tissues of patients enrolled in the prospective trial allowed a successive investigation of the role of *EGFR* mutational status, both in ECD and TKD, and the *EGFR* amplification. We demonstrated that 6 patients harbored mutations in *EGFR* ECD, none of them previously described. Four mutated patients were enrolled in the P-GEMOX arm; their PFS ranged from 2 to 9 months and the OS between 5.7 to 15.2 months. The survival curves showed that *EGFR* ECD mutations may have a predictive negative role in patients treated with panitumumab, but considering the worst survival of ICC patients having *EGFR* ECD mutations as compared to WT, the impact of *EGFR* ECD mutations may be rather prognostic, at least in this site subgroup.

Seven out of 57 patients harbored mutations in the exons 18–21 of *EGFR*, coding for the TKD: of them, two were novel mutations, while the others were already described in literature in other cancer types [25–28]. In particular, the V786M is described as high sensitive to gefitinib treatment in bronchioloalveolar adenocarcinoma [29]. Interestingly, none of *EGFR* TKD mutated patients had a tumor response, even if the correlation is not statistically significant (p = 0.07, data not shown). Even if not affecting the survival in the entire cohort, *EGFR* TKD mutated patients treated with P-GEMOX had a worse prognosis compared to WT patients.

Although the biological impact of each of the molecular alterations investigated may be different, we have considered all the mutations affecting *EGFR*, *NRAS*, *KRAS*, *BRAF*, and *PIK3CA* as potentially involved in the resistance to anti-EGFR therapies in BTC. For this reason, we evaluated the effect of treatments in patients without any of the mutations, referred to as “quintuple negative” population. This population does not seem to have different prognosis, and received similar benefit with both GEMOX and P-GEMOX as compared with patients with any of the mutations. By subgroup analysis, ECC quintuple negative patients have a trend towards a better survival than mutated patients, whereas in other site subgroups, mutation analysis revealed no prognostic impact.

Thirty-seven patients had tumor sample available for *EGFR* amplification analysis by FISH. More than half of the tested samples resulted FISH+. *EGFR* was amplified in all site subgroups (ICC, ECC, and GBC). Overall, *EGFR* amplification did not correlate with PFS or OS. According to the site of the disease, we found that *EGFR* amplification significantly correlated with poor PFS in GBC patients, while in ECC there was a trend towards a better survival of FISH+. Of note, 18 out of 19 *EGFR* amplified patients had lymph-node infiltration, suggesting a more advanced disease. This finding is enforced by a work on gastroesophageal cancers, in which *EGFR* amplification seemed to correlate with worse prognosis and lymph node metastasis [30]. However, the role of *EGFR* amplification as prognostic biomarker remains controversial. In two studies in mCRC, authors demonstrated that *EGFR* amplification was associated with longer PFS and OS [31, 32]; otherwise, in another work, *EGFR* amplification was not associated to prognosis [17].

The EGFR protein expression was not evaluated in our case series; previous studies demonstrated that EGFR is expressed in about 60% of BTC, ranging from 54% to 65%, and that the EGFR positivity does not affect the clinical outcome, independently from the treatment and site of origin [9, 23, 24, 33]. In contrast, Yang and collaborators evaluated the EGFR expression in 175 BTC, demonstrating that EGFR expression is a negative prognostic factor in ICC, but not in ECC [19, 34]. Therefore, the role of EGFR expression remains controversial, mainly due to the high heterogeneity of tumors analyzed.

The Vecti-BIL study included all *KRAS* exon 2 WT patients, but no other molecular stratification was performed at the time of enrollment, yielding to a highly heterogeneous population both in terms of site subgroups and of molecular alterations. Even if patients were selected for the absence of *KRAS* mutation, the antitumor effect of panitumumab was demonstrated neither in the overall population, nor in the molecular subgroups in which *EGFR* pathway was deemed suitable for effective inhibition. Most of the genetic alterations found seem to have a prognostic significance rather than a predictive role towards the anti-EGFR therapy. A high molecular heterogeneity seems to be a predominant feature of BTC, preventing the identification of well-defined subgroup of patients, which could benefit from targeted therapies. Due to the small cohort of patients analyzed, we can only speculate about prognostic or predictive role of EGFR status; nevertheless, the meta-analysis conducted by Chen and collaborators [35], which pooled four studies to examine the efficacy of anti-EGFR therapies combined to standard chemotherapy, revealed promising results of EGFR-targeted therapy in increasing the survival rate (PFS) of advanced BTC patients. These data keep the door open to the anti-EGFR therapies, provided that an accurate selection of patients is carried out. Moreover, it is highly recommended the identification of other biomarkers able to select *a priori* subgroups of patients who could benefit from these treatments. One of the suggested predictive biomarkers of response/resistance to anti-EGFR therapies is the expression of EGFR ligands; Luraghi and collaborators demonstrated that NRG1 is a marker of resistance to cetuximab, while EREG and AREG expression does not confer resistance to anti-EGFR therapies in mCRC xenospheres [36]. Further, another proposed mechanism is the presence of a specific oncogenic variant of EGFR, the EGFRvIII, which is constitutively active, without the requirement of ligand binding; in fact, it has been demonstrated that anti-EGFR antibodies are less effective in the presence of this variant in glioblastoma multiforme [37]. EGFR promoter methylation could be involved in the lack of response to anti-EGFR antibodies; in the work of Scartozzi, it has been demonstrated that mCRC patients treated with cetuximab displaying EGFR promoter methylation are less responder compared to unmethylated patients, with a significant worse prognosis [38]. This is only an overview of the main mechanisms involved in pathogenesis and drug resistance in BTC, evidencing the complexity of EGFR pathway and the major points of failure of anti-EGFR therapies. In fact, EGFR activation could be sustained also by a defective ubiquitination and subsequent internalization, which can cause a prolonged activation as well as a transactivation through COX-2/PgE2 signaling, as shown by Yoon and collaborators in BTC cell lines [39].

Since BTCs are rare disease, great efforts have been made in recent years in conducting prospective studies to investigate the efficacy of new therapies. Standard of care has been identified in advanced disease [40, 41] and a variety of targeted therapies has been tested so far [11, 12, 42–44]. The inclusion of patients with primary cancers arising in different part of the biliary tree is a compromise generally accepted in clinical trials. However, with the advent of new technologies as next-generation sequencing, it has become evident that mutational profiling of BTC varies within the tumor location. As a consequence, a neglected genetic heterogeneity can result in findings difficult to interpret. Thus, it is desirable that the genomic landscape of BTC will take the place of pathologic criteria based on the site of origin in future trials [45].

## Supporting information

S1 Fig Kaplan-Meier survival curves in *EGFR* or its transducers mutated vs WT patients. A-B) association between the presence of any mutations and PFS and OS, respectively, in ICC patients. C-D) association between the presence of any mutation and PFS and OS, respectively, in ECC patients. E-F) association between the presence of any mutation and PFS and OS, respectively, in GBC patients.
(TIF)

S1 Table Summary of mutations found in 57 BTC patients. (DOCX)
