# Supplementary figures and images for: Prognostic and predictive role of EGFR pathway alterations in biliary cancer patients treated with chemotherapy and anti-EGFR
Source: PLoS One. 2018 Jan 19;13(1):e0191593. doi: 10.1371/journal.pone.0191593 (PMC5774843; doi:10.1371/journal.pone.0191593)

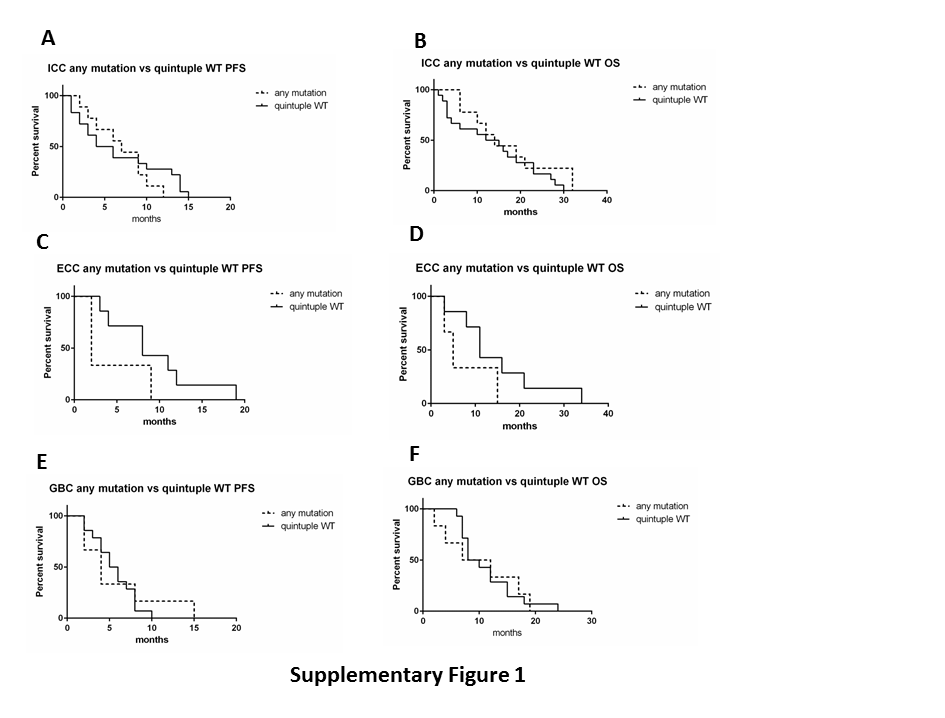

Supplement: S1 Fig — A-B) association between the presence of any mutations and PFS and OS, respectively, in ICC patients. C-D) association between the presence of any mutation and PFS and OS, respectively, in ECC patients. E-F) association between the presence of any mutation and PFS and OS, respectively, in GBC patients. (TIF) [file pone.0191593.s001.tif]
