# Supplementary material for: Prognostic and predictive role of EGFR pathway alterations in biliary cancer patients treated with chemotherapy and anti-EGFR
Source: PLoS One. 2018 Jan 19;13(1):e0191593. doi: 10.1371/journal.pone.0191593 (PMC5774843; doi:10.1371/journal.pone.0191593)
Supplement: S1 Table — (DOCX) [file pone.0191593.s002.docx]

**TABLE S1**. Summary of mutations found in 57 BTC patients

| **Patient ID** | ***EGFR* ECD**  **mutations** | ***EGFR* TKD mutations** | **Previous description**  **(cancer type)** | **PMID** |
| --- | --- | --- | --- | --- |
| #10101 | G482E |  |  | Novel mutation |
| #40733 | L469S |  |  | Novel mutation |
| #70636 | L443Q |  |  | Novel mutation |
| #71772 | K467stop/N468D |  |  | Novel mutations |
| #71878 | G482R/S464P |  |  | Novel mutation |
| #90218 | N468D | G824S |  | Novel mutations |
| #70215 |  | L707S | Colon | 22026926 |
| #30276 |  | V786M | Lung, prostate, breast | 16567021/18379371/24406864/24595526 |
| #40106 |  | L788H/ T854I | Lung | Novel mutation/21409490 |
| #70113 |  | G810S | Lung | 16014883/156567021 |
| #40322 |  | D855N | Lung | 19724844/19088172 |
| #50109 |  | D837N | Lung | 23749122 |
